# Association between serum glucose/potassium ratio and acute kidney injury in patients with traumatic brain injury: based on MIMIC-IV database

**DOI:** 10.7189/jogh.16.04108

**Published:** 2026-03-27

**Authors:** Yi Chen, Shuang Li, Chongbin Zhang, Xianliang Zeng

**Affiliations:** 1 Emergency Comprehensive Ward Electronic Intensive Care Unit, Huizhou First Hospital, Huizhou, China; 2 Department of Emergency Surgery, Huizhou First Hospital, Huizhou, China

## Abstract

**Background:**

Acute kidney injury (AKI) is a common complication in patients with traumatic brain injury (TBI) and is closely linked to prognosis. Although the serum glucose-to-potassium ratio (GPR) is a prognostic indicator in various diseases, its clinical significance in this specific patient population remains unexplored.

**Methods:**

We identified patients requiring intensive care unit admission from the critical care medical information database and stratified them into quintiles based on GPR. The nonlinear relationship between GPR and AKI in TBI patients was analysed by restricted cubic splines (RCS). We conducted an exploratory subgroup analysis to investigate the differences in this association across different subgroups. Using mediation analysis, we aimed to explore the potential mediating effect of fluid balance in the development of AKI in patients with GPR and TBI.

**Results:**

1536 patients with TBI were included, of whom 1162 developed AKI. Every one unit increase in GPR increases the risk of AKI in TBI patients by 1.3% (odds ratio (OR) = 1.013; 95% confidence interval CI) = 1.004–1.024, *P* = 0.014). Further, RCS analysis indicated an approximately linear positive correlation between the increase in GPR and the AKI risk (*P* = 0.235). Subgroup analysis indicated that both patients with abnormal serum glucose and those with normal potassium levels showed a significant positive correlation with AKI risk (OR>1, *P* < 0.05). Fluid balance potentially mediated the association between GPR and TBI-induced AKI in part, with a mediating effect of 4.49 × 10^−4^ (95% CI = 2.04 × 10^−4^–1.18 × 10^−3^, *P* < 0.001).

**Conclusions:**

GPR is significantly associated with AKI risk in TBI patients, which may be mediated by FB. We identified GPR as a new biomarker for assessing AKI risk in TBI patients, thereby offering fresh insights into prevention and treatment for this population.

Traumatic brain injury (TBI) is one of the leading causes of death and disability worldwide, with 27.16 million cases and 48.99 million prevalent cases in 2019, as reported by the Global Disease Burden database [1,2]. TBI patients are often accompanied by a variety of complications, of which acute kidney injury (AKI) is one of the most prevalent and severe complications, with the incidence rate ranging from 17–20% [3,4]. AKI is not only associated with the severity of TBI, but also dramatically impacts patients’ long-term survival rates, hospital stays, and the risk of multiple organ failure [5,6]. Therefore, early identification of patients with high-risk AKI is essential to improve the prognosis.

Abnormal serum glucose and potassium metabolism have been shown to correlate with AKI, with elevated serum glucose and hypokalaemia serving as independent predictors of AKI [7,8]. The serum glucose/potassium ratio (GPR), an emerging biomarker, reflects the link between metabolic disturbances and renal dysfunction and has been proposed as a biological marker for various pathophysiological conditions, such as TBI, acute spinal cord injuries, and cerebrovascular haemorrhage [9–11]. However, the GPR function in TBI patients with AKI is unexplored.

We aimed to analyse the correlation between GPR and AKI risk in TBI patients using the Medical Information Mart for Intensive Care-IV (MIMIC-IV) database, thereby identifying a powerful marker to aid risk stratification for TBI patients with AKI and providing new ideas and evidence for clinical intervention.

## METHODS

### Database

This retrospective analysis was based on the MIMIC-IV database, version 2.2 (Beth Israel Deaconess Medical Centre, Boston, Massachusetts, USA), which housed the clinical data of patients between 2008 and 2019 and stored a vast amount of clinical information, including demographic characteristics, vital signs, urine output, underlying diseases, disease severity assessments, laboratory information, medications, and interventions [12]. MIMIC-IV is an open and anonymous database that contains no sensitive information about the samples. Therefore, no approval from the local ethics committee is required.

### Inclusion and exclusion criteria

Identification of patients with TBI in the MIMIC-IV database was based on the International Classification of Diseases (ICD) Ninth Revision code 85 and ICD-10th revision code S06 [13]. We screened 2316 TBI patients admitted to the intensive care unit (ICU). The diagnostic criteria for AKI were based on the Kidney Disease: Improving Global Outcomes Guidelines [14,15]. The criteria were as follows: stage one (serum creatinine (SCr) increased 1.5–1.9 times from baseline or by ≥0.3 mg/dL, or urine output <0.5 mL/kg/h for six to 12 hours); stage two: SCr increased 2.0–2.9 times from baseline, or urine output <0.5 mL/kg/h for ≥12 hours); stage three (SCr increased ≥3.0 times from baseline, or SCr ≥4.0 mg/dL with an acute increase, or urine output <0.3 mL/kg/h for 24 hours, or anuria for ≥12 hours). We defined baseline creatinine as the lowest SCr value within the seven days before ICU admission. The baseline estimated glomerular filtration rate (eGFR) was calculated using the chronic kidney disease-epidemiology formula. Early AKI was defined as AKI occurring within ≤48 hours after ICU admission, while late AKI referred to events occurring >48 hours after ICU admission [16].

**Figure 1:**
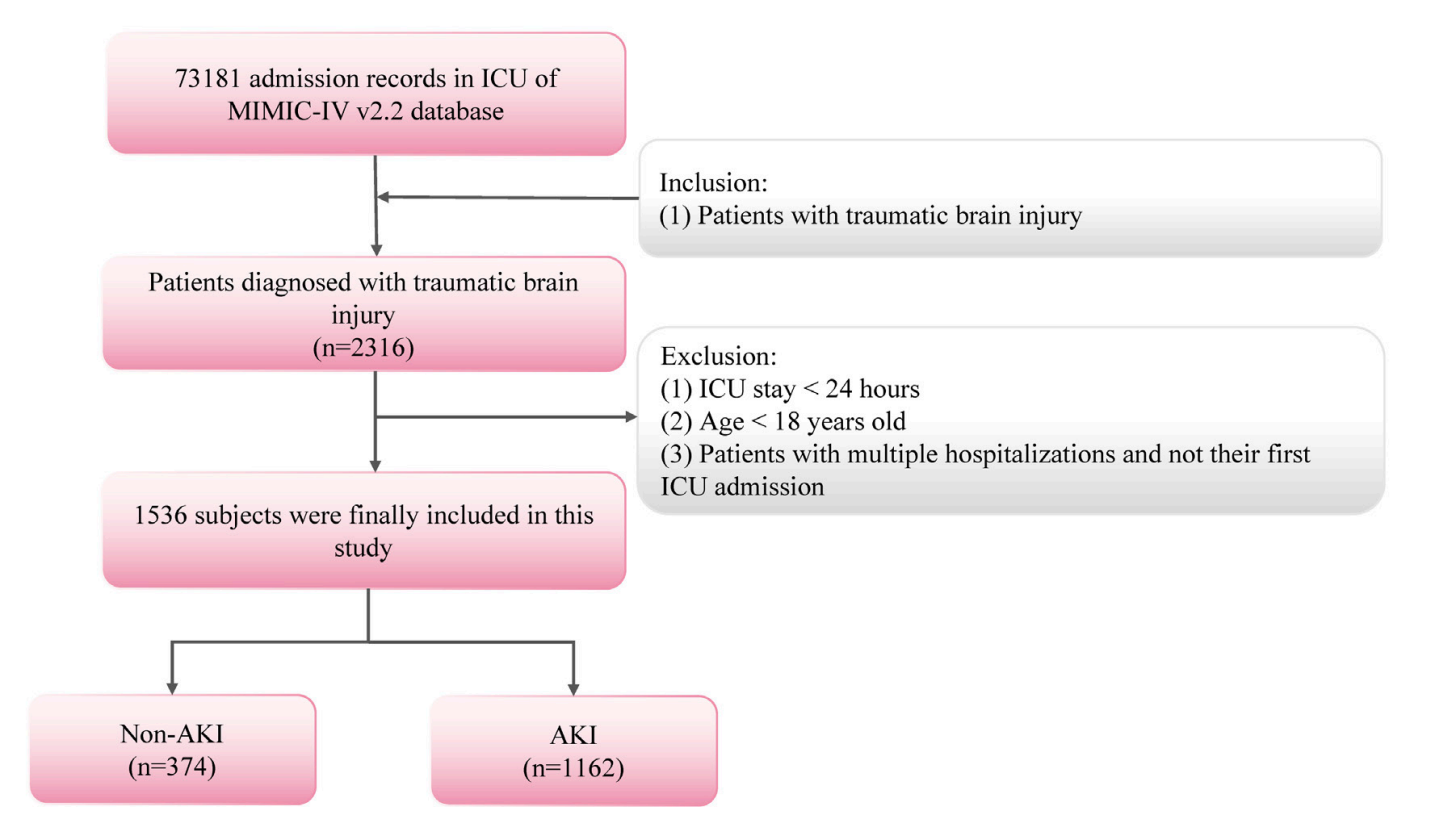
Inclusion and exclusion criteria.

Patients with any of the following conditions were excluded: those with an ICU stay of <1 day, those aged <18 years, and those with multiple hospitalisations and non-first ICU admissions. A total of 1536 eligible patients were enrolled in the investigation (**Figure 1**).

### Data collection

We calculated the GPR by the division operation of glucose and potassium levels [17]. GPR was calculated based on the first recorded serum glucose and potassium measurements within 24 hours after the first admission to the ICU (GPR = serum glucose (mg/dL)/serum potassium (mmol/L)) [18]. In addition, by evaluating the contribution of two components (serum glucose and potassium) to the risk of AKI in TBI patients, we divided patients into a normal serum glucose group (70–100 mg/dL) and an abnormal group, as well as a normal potassium group (3.5–5.5 mEq/L) and an abnormal group.

Candidate covariates were selected based on clinical relevance and supporting literature [19,20]. Specifically, we included factors known to be closely associated with TBI prognosis or AKI risk: demographic characteristics (age, sex, and race), comorbidities (diabetes, chronic kidney disease, myocardial infarction, cerebrovascular disease, and sepsis, injury severity scores (Glasgow Coma Scale (GCS), and TBI severity classification), early ICU vital signs and laboratory values (mean blood pressure), body temperature, platelet count, serum sodium, serum potassium, blood glucose, blood urea nitrogen (BUN), creatinine, prothrombin time (PT), international normalised ratio (INR), and red blood cell distribution width (RDW), baseline renal function (eGFR) calculated using the chronic kidney disease-epidemiology formula to adjust for baseline kidney function differences, and treatment measures (vasopressor use, hyperosmolar therapy, anticoagulation, invasive mechanical ventilation, fluid resuscitation, diuretics, glucocorticoids, and vancomycin). Severity scores, vital signs, and biochemical indicators collected multiple times during the ICU stay were all determined by the values corresponding to the most severe level measured within the first 24 hours after ICU admission. The primary outcome was AKI during the ICU.

TBI patients often experience fluid balance (FB) disorders due to factors such as stress response and dehydration treatment, with early fluid status changes being closely associated with patient prognosis. Therefore, we considered fluid overload (FO) occurring on the first day of ICU admission as a secondary outcome. FB was defined as the total fluid input minus the total fluid output on the first day of ICU admission. The Fluid Balance Index (FBI) was computed using the following formula: FBI = (total liquid input – total liquid output)/initial body weight (kg). FO was defined as the accumulation of FBI exceeding 10% of the initial body weight [21].

### Statistical analysis

We constructed a baseline table using the ‘tableone’ package and conducted a skewness test on continuous variables. Data with a severe skewness distribution (mean/standard deviation (SD)<2) was represented by median and interquartile range, and we used the Wilcoxon rank sum test for intergroup comparison. We summarised non-severely skewed data as mean (SD), and used the *t* test for intergroup comparisons. Classification data are presented as n (%), and we used the χ^2^ test for intergroup comparisons. A bilateral *P*-value <0.05 indicated significant differences. We calculated the variance inflation factor (VIF) to assess collinearity among variables, and collinearity was considered present when VIF>4 (Table S1 in the **Online Supplementary Document**). AKI was regarded as a binary outcome. Given that the proportional hazards assumption was violated in the Cox proportional hazards model, we used a multivariate logistic regression to estimate the association between GPR and AKI. By integrating the results of multicollinearity tests and constructing a logistic regression model, we fine-tuned the model to investigate the relationship between GPR and AKI in TBI patients. We grouped the respondents into quintiles of GPR and constructed a logistic regression model of GPR quintiles, including FO and AKI, in TBI patients.

To assess the robustness of the main findings and the incremental prognostic value of GPR, we conducted several pre-specified sensitivity and additional analyses. For AKI definition and classification, we included only patients with documented creatinine values within seven days prior to ICU admission, based on which AKI was redefined to assess its association with GPR. We further performed stratified analyses according to AKI stage and timing (early/late onset) to examine association patterns across AKI subtypes.

Regarding GPR dynamics and time dimensions, we calculated the mean GPR values for 0–24, 24–48, and 48–72 hours after admission. AKI was treated as a time-to-event outcome in survival analyses. We compared the effects and model performance of 72-hour mean GPR with baseline single-point GPR, with adjustments for the coefficients of variation of blood glucose and potassium to assess robustness.

To control for confounding and bias, we conducted sensitivity analyses to examine the influence of insulin use. Additionally, we excluded patients receiving renal replacement therapy (RRT) during their ICU stay to mitigate bias in treatment decisions.

Regarding incremental predictive value, we constructed hierarchical adjusted models using traditional risk factors (including blood glucose and potassium), and we compared the performance of models with and without GPR.

In the regression model adjusting for all confounding factors, we explored the nonlinear relationship between GPR, FO, and AKI in TBI patients using restricted cubic splines (RCS). We used subgroup analysis to explore the specificity of the subgroups, and applied the Bonferroni correction to control the risk of false positives. Additionally, we assessed whether the differences in effects across subgroups were statistically significant by including interaction terms and calculating interaction *P*-values. In conclusion, to explore the potential role of FB in the association between GPR and AKI in patients with TBI, we constructed a mediation model. The crude model had no adjustment. Model 1 had adjustments for age, race, and sex and model 2 had adjustments on all variables.

We collected data from the MIMIC-IV (version 2.2) database using the Structured Query Language and *R*, version 4.4.1 (R Core Team, Vienna, Austria) for data analysis. The *R* packages used included ‘mice,’ ‘tableone,’ ‘survival,’ ‘rms,’ and ‘mediate.’ The bilateral *P* < 0.05 was considered statistically significant. We excluded variables with missing values exceeding 20% of the total sample size in life characteristics and biochemical indicators (Figure S1 in the **Online Supplementary Document**) and handled other missing variables using the random forest method.

## RESULTS

### Baseline characteristics

We enrolled 1536 patients with TBI, of whom 1162 presented with AKI (**Table 1**). Males comprised 61.0% of the total, with an average age at admission of 65.21 years (SD = 20.74). Compared with the non-AKI group, in the AKI group, the levels of GPR, blood glucose, serum potassium, BUN, RDW, creatinine, INR, PT, FB, and FBI, the percentages of RRT, fluid resuscitation, and FO, and ICU mortality were higher, while use of diuretics and the baseline eGFR were lower (*P* < 0.05).

**Table 1:** Baseline characteristics of included patients at the first ICU admission*

Characteristics	Total (n = 1536)	Non-AKI (n = 374)	AKI (n = 1162)	*P*-value
GPR, MD (IQR)	31.30 (25.35–39.54)	29.76 (24.74–37.57)	31.81 (25.71–40.48)	0.004
Sex				0.074
*Female*	599 (39.0)	161 (43.0)	438 (37.7)	
*Male*	937 (61.0)	213 (57.0)	724 (62.3)	
Age in years, x̄ (SD)	65.21 (20.74)	62.53 (22.53)	66.07 (20.06)	0.004
Race				0.950
*White*	951 (61.9)	232 (62.0)	719 (61.9)	
*Black*	83 (5.4)	19 (5.1)	64 (5.5)	
*Other race*	502 (32.7)	123 (32.9)	379 (32.6)	
BP in mmHg, x̄ (SD)	81.57 (10.13)	81.24 (9.43)	81.68 (10.35)	0.473
Body temperature in °C, x̄ (SD)	37.02 (0.50)	37.00 (0.44)	37.03 (0.52)	0.363
Glucose in mg/dL, MD (IQR)	126.00 (104.00–159.00)	119.00 (101.00–141.00)	128.00 (105.00–165.00)	<0.001
GCS, x̄ (SD)	13.07 (2.65)	13.09 (2.52)	13.07 (2.69)	0.916
Platelet in K/μL, x̄ (SD)	181.03 (83.61)	187.88 (81.25)	178.82 (84.28)	0.068
Potassium in mmol/L, x̄ (SD)	4.09 (0.71)	4.00 (0.59)	4.12 (0.74)	0.005
Sodium in mEq/L, x̄ (SD)	137.78 (4.90)	137.76 (4.87)	137.78 (4.91)	0.942
BUN in mg/dL, MD (IQR)	17.00 (12.00–24.00)	16.00 (11.00–22.00)	18.00 (13.00–25.00)	<0.001
RDW, x̄ (SD)	14.38 (1.97)	14.07 (1.83)	14.47 (2.00)	0.001
Creatinine in mg/dL, MD (IQR)	0.90 (0.80–1.20)	0.90 (0.70–1.10)	1.00 (0.80–1.20)	<0.001
INR, MD (IQR)	1.20 (1.10–1.30)	1.10 (1.10–1.30)	1.20 (1.10–1.40)	<0.001
PT(s), MD (IQR)	13.00 (11.90–14.80)	12.80 (11.70–14.00)	13.00 (11.90–15.00)	0.001
Baseline eGFR, x̄ (SD)	77.72 (28.28)	81.05 (27.92)	76.65 (28.33)	0.009
Invasive ventilation				<0.001
*No*	888 (57.8)	249 (66.6)	639 (55.0)	
*Yes*	648 (42.2)	125 (33.4)	523 (45.0)	
Vasopression				
*No*	1510 (98.3)	370 (98.9)	1140 (98.1)	0.399
*Yes*	26 (1.7)	4 (1.1)	22 (1.9)	
Hyperosmolar therapy				
*No*	1445 (94.1)	360 (96.3)	1085 (93.4)	0.054
*Yes*	91 (5.9)	14 (3.7)	77 (6.6)	
Anticoagulant therapy				
*No*	1211 (78.8)	293 (78.3)	918 (79.0)	0.842
*Yes*	325 (21.2)	81 (21.7)	244 (21.0)	
Chronic kidney disease				
*No*	1361 (88.6)	341 (91.2)	1020 (87.8)	0.088
*Yes*	175 (11.4)	33 (8.8)	142 (12.2)	
Sepsis				
*No*	1495 (97.3)	367 (98.1)	1128 (97.1)	0.360
*Yes*	41 (2.7)	7 (1.9)	34 (2.9)	
Myocardial infarction				0.093
*No*	1412 (91.9)	352 (94.1)	1060 (91.2)	
*Yes*	124 (8.1)	22 (5.9)	102 (8.8)	
Cerebrovascular disease				0.061
*No*	1366 (88.9)	343 (91.7)	1023 (88.0)	
*Yes*	170 (11.1)	31 (8.3)	139 (12.0)	
Diabetes				0.051
*No*	1378 (89.7)	346 (92.5)	1032 (88.8)	
*Yes*	158 (10.3)	28 (7.5)	130 (11.2)	
TBI severity				0.357
*Mild*	957 (62.3)	224 (59.9)	733 (63.1)	
*Moderate*	452 (29.4)	121 (32.4)	331 (28.5)	
*Severe*	127 (8.3)	29 (7.8)	98 (8.4)	
RRT				<0.001
*No*	1495 (97.3)	374 (100.0)	1121 (96.5)	
*Yes*	41 (2.7)	0 (0.0)	41 (3.5)	
Diuretics				0.003
*No*	1455 (94.7)	366 (97.9)	1089 (93.7)	
*Yes*	81 (5.3)	8 (2.1)	73 (6.3)	
Glucocorticoids				0.962
*No*	1473 (95.9)	358 (95.7)	1115 (96.0)	
*Yes*	63 (4.1)	16 (4.3)	47 (4.0)	
Vancomycin				0.674
*No*	1411 (91.9)	346 (92.5)	1065 (91.7)	
*Yes*	125 (8.1)	28 (7.5)	97 (8.3)	
Fluid resuscitation				0.020
*No*	173 (11.3)	55 (14.7)	118 (10.2)	
*Yes*	1363 (88.7)	319 (85.3)	1044 (89.8)	
FB in mL, MD (IQR)	2888.30 (1064.88–5519.38)	1902.70 (529.70–3655.63)	3275.33 (1267.38–6126.22)	<0.001
FBI in mL/kg, MD (IQR)	40.36 (14.60–77.75)	29.09 (8.02–58.73)	44.15 (17.20–83.98)	<0.001
FO				<0.001
*No*	1280 (83.3)	334 (89.3)	946 (81.4)	
*Yes*	256 (16.7)	40 (10.7)	216 (18.6)	
ICU mortality				0.002
*No*	1416 (92.2)	359 (96.0)	1057 (91.0)	
*Yes*	120 (7.8)	15 (4.0)	105 (9.0)	

AKI – acute kidney injury, BP – blood pressure, BUN – blood urea nitrogen, eGFR – estimated glomerular filtration rate, FBI – fluid balance index, FO – fluid overload, GCS – Glasgow Coma Scale, GPR – glucose-potassium ratio, ICU – intensive care unit, INR – international normalised ratio, IQR – interquartile range, MD – median, PT – prothrombin time, RDW – red blood cell distribution width, RRT – renal replacement therapy, SD – standard deviation, TBI – traumatic brain injury, x̄ – mean

*Presented as n (%) unless specified otherwise.

### Association between GPR and clinical outcomes in TBI patients

#### Association of GPR with AKI and FO in TBI patients

When GPR was analysed as a continuous variable, for every one unit increase in GPR, the risk of FO in TBI patients increased by 0.6% (odds ratio (OR) = 1.006; 95% confidence interval (CI) = 1.001–1.014, *P* = 0.041), and the AKI risk increased by 1.3% (OR = 1.013; 95% CI = 1.004–1.024, *P* = 0.014). The association was statistically significant. When GPR was analysed as a categorical variable, after adjusting for all confounding factors, compared to the lowest quartile group (Q1), Q2 (OR = 1.675; 95% CI = 1.036–2.731, *P* = 0.037) and Q5 (OR = 2.592; 95% CI = 1.590–4.276, *P* < 0.001) showed that the GPR levels were associated with an increased risk of FO, with the increase rates being 67.5% and 159.2%, respectively (**Table 2**).

**Table 2:** Association between GPR, fluid overload, and AKI in patients with TBI*

Characteristics	Crude model, OR (95% CI)	*P*-value	Model 1, OR (95% CI)	*P*-value	Model 2, OR (95% CI)	*P*-value
**FO**						
GPR (continuous)	1.007 (1.003–1.013)	0.006	1.008 (1.003–1.014)	0.007	1.006 (1.001–1.014)	0.041
GPR (categorical)						
*Q1 (<24.200)*	ref.		ref.		ref.	
*Q2 (24.200–28.857)*	1.522 (0.983–2.373)	0.061	1.474 (0.936–2.338)	0.096	1.675 (1.036–2.731)	0.037
*Q3 (28.857–34.444)*	1.037 (0.649–1.657)	0.880	1.054 (0.649–1.711)	0.833	1.115 (0.666–1.869)	0.680
*Q4 (34.444–42.581)*	1.181 (0.748–1.870)	0.476	1.469 (0.913–2.374)	0.114	1.507 (0.905–2.520)	0.116
*Q5 (≥42.581)*	2.053 (1.350–3.160)	<0.001	2.438 (1.569–3.836)	<0.001	2.592 (1.590–4.276)	<0.001
**AKI**						
GPR (continuous)	1.016 (1.007–1.026)	0.001	1.016 (1.006–1.026)	0.002	1.013 (1.004–1.024)	0.014
GPR (categorical)						
*Q1 (<24.200)*						
*Q2 (24.200–28.857)*	0.37 (0.658–1.334)	0.719	0.930 (0.651–1.327)	0.687	0.964 (0.669–1.389)	0.844
*Q3 (28.857–34.444)*	1.199 (0.833-1.729)	0.330	1.200 (0.832–1.736)	0.330	1.230 (0.842–1.798)	0.285
*Q4 (34.444–42.581)*	1.141 (0.795–1.641)	0.475	1.076 (0.746–1.553)	0.696	1.006 (0.689–1.471)	0.974
*Q5 (≥42.581)*	1.618 (1.106–2.379)	0.014	1.598 (1.086–1.766)	0.017	1.442 (0.957–2.184)	0.081

AKI – acute kidney injury, CI – confidence interval, FO – fluid overload, GPR – glucose-potassium ratio, OR – odds ratio, ref – reference, Q – quantile

*The crude model was unadjusted. Model 1 had adjustments for age, race, and sex. Model 2 had adjustment for age, race, sex, mean blood pressure, body temperature, platelet count, sodium ion concentration, mean red blood cell width, creatinine level, prothrombin time, international normalised ratio, baseline estimated glomerular filtration rate, Glasgow Coma Scale score, vasopressin, hypertonic therapy, anticoagulant therapy, sepsis, chronic kidney disease, myocardial infarction, cerebrovascular disease, diabetes, fluid resuscitation, diuretics, glucocorticoids, and vancomycin.

#### Sensitivity analysis

To evaluate the robustness of the main findings, we conducted a series of sensitivity analyses. Reanalysis of baseline characteristics with recorded creatinine confirmed the association between GPR and AKI risk, supporting the robustness of the primary findings regarding AKI definition (Table S2 in the **Online Supplementary Document**). Stratified analyses by AKI stage and time of onset showed that, compared to the reference group, GPR was significantly and positively associated with the risk of stage two AKI, stage three AKI, and early and late AKI (Table S3 in the **Online Supplementary Document**).

Regarding GPR dynamics and model performance, analysis over 0–72 hours showed that higher mean GPR values were consistently and significantly associated with increased AKI risk (OR>1, *P* < 0.05), aligning with the primary analysis based on a single GPR value within 24 hours of admission (Table S4 in the **Online Supplementary Document**). Cox regression analysis treating AKI as a time-to-event outcome confirmed that higher GPR levels remained significantly associated with increased AKI risk (Table S5 in the **Online Supplementary Document**). Exploratory analysis revealed significant associations in both the 72-hour mean GPR and baseline single GPR models, with highly consistent effect directions. Model comparison indicated a slightly lower Akaike information criterion (AIC) for the mean GPR model and a marginally increased area under the receiver operating characteristic curve from 0.645 to 0.650 (Table S6 in the **Online Supplementary Document**). The association between GPR and AKI remained highly robust after adjusting for the variation coefficients of blood glucose and potassium, as in the original model (Table S7 in the **Online Supplementary Document**).

The model adjusted for insulin use demonstrated that GPR remained significantly and positively associated with AKI risk (Table S8 in the **Online Supplementary Document**). After excluding patients who received RRT, the direction and significance of the associations between GPR and both FO and AKI remained unchanged (OR 1, *P* < 0.05), supporting the robustness of the main findings (Table S9 in the **Online Supplementary Document**).

To evaluate the incremental prognostic value of GPR, we systematically compared the predictive performance of different models. In a model adjusting for both blood glucose and potassium, GPR remained significantly and independently associated with AKI risk. The model comparison showed improved fit upon adding GPR, as indicated by a decrease in AIC from 1682.1 to 1679.6. Taken together, the improvement in model fit and the sustained independent effect of GPR after multiple adjustments support the incremental value of GPR (Table S10 in the **Online Supplementary Document**).

#### Association of GPR with the risk of AKI and FO in TBI patients

**Figure 2:**
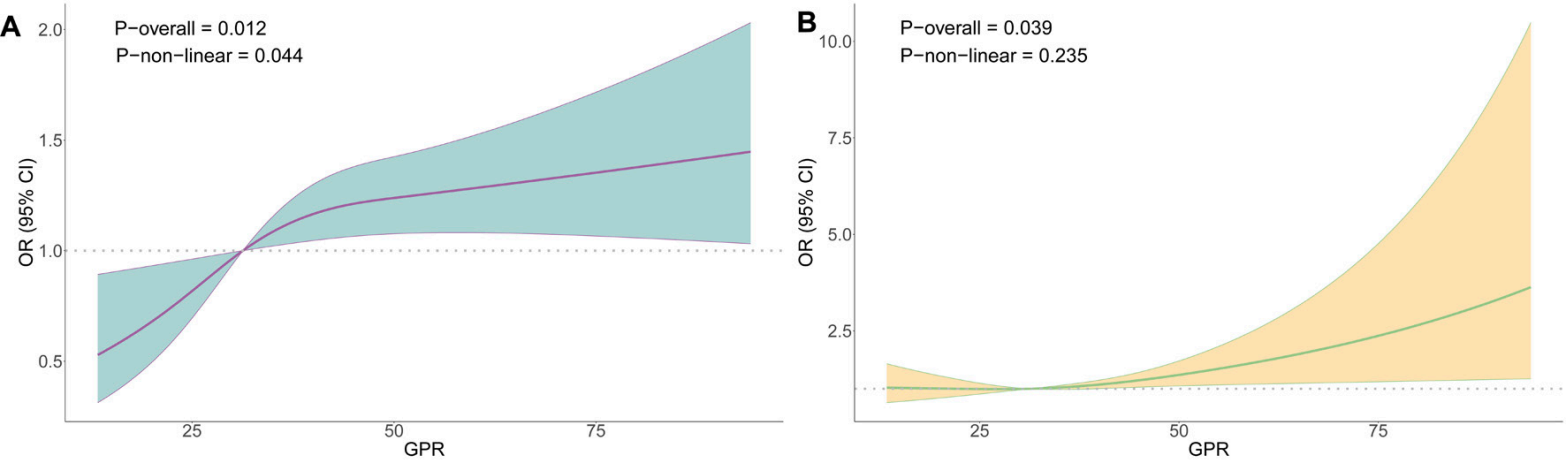
ROC curves between GPR and fluid overload. **Panel A.** TBI patients. **Panel B.** Between GPR and AKI in TBI patients.

After adjusting for all potential confounding factors, RCS analysis revealed a significant nonlinear trend between GPR and the risk of FO in TBI patients (overall *P* = 0.012 and nonlinear *P* = 0.044) (**Figure 2**, Panel A). Additionally, we observed a significant trend between GPR and the AKI risk in TBI patients (*P* = 0.039), but the association between the two was close to linear (nonlinear *P* = 0.235) (**Figure 2**, Panel B).

#### Exploratory subgroup analysis

**Figure 3:**
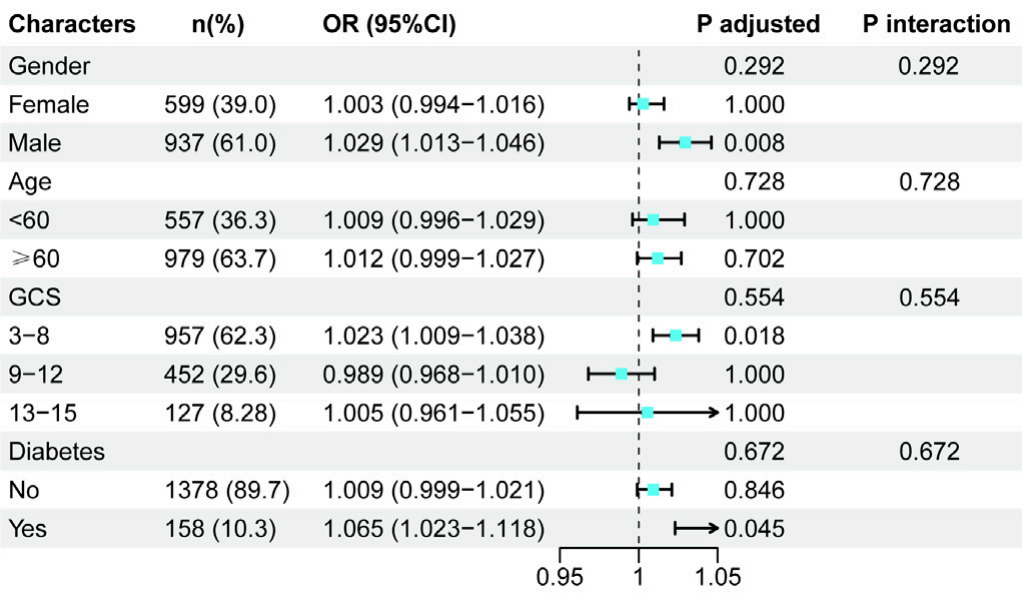
Subgroup analysis (after Bonferroni correction).

To confirm the stability of our results, we also conducted exploratory subgroup analysis stratified by age, sex, GCS, and diabetes. The results showed that the association between GPR and the risk of AKI in TBI patients was statistically significant only in males (OR = 1.029; 95% CI = 1.013–1.046, *P* = 0.008), GCS≤8 (OR = 1.023; 95% CI = 1.009–1.038, *P* = 0.018), and participants with diabetes (OR = 1.065; 95% CI = 1.023–1.118, *P* = 0.045). The analysis for interaction effects did not reveal any significant effect modification by sex (*P* = 0.292), GCS score (*P* = 0.728), or diabetes status (*P* = 0.672) on the association involving GPR (**Figure 3**).

In addition, we also evaluated the contribution of changes in two components of GPR (serum glucose and potassium) to the risk of AKI in TBI patients (**Table 3**). When GPR was treated as a continuous variable, serum glucose (OR = 1.005; 95% CI = 1.002–1.008, *P* < 0.001) and serum potassium (OR = 1.248; 95% CI = 1.029–1.529, *P* = 0.028) were significantly and positively correlated with AKI risk. When GPR was converted as a categorical variable, in the model adjusted for all confounding factors, patients with abnormal blood glucose (OR = 1.015; 95% CI = 1.004–1.027, *P* = 0.017) and patients with normal serum potassium (OR = 1.015; 95% CI = 1.002–1.028, *P* = 0.030) were significantly associated with AKI risk.

**Table 3:** Independent association between normal/abnormal grouping of glucose or potassium and AKI risk in TBI patients

Characteristics	n (%)	OR (95% CI)	*P*-value
Glucose (continuous)		1.005 (1.002–1.008)	<0.001
Normal glucose (categorical)			
*No*	1244 (81.0)	1.015 (1.004–1.027)	0.017
*Yes*	292 (19.0)	1.023 (0.944–1.110)	0.584
Potassium (continuous)		1.248 (1.029–1.529)	0.028
Normal potassium (categorical)			
*No*	252 (16.4)	1.013 (0.998–1.035)	0.229
*Yes*	1284 (83.6)	1.015 (1.002–1.028)	0.030

CI – confidence interval, OR – odds ratio

#### Mediating effect analysis

In the mediation analysis, we explored the potential role of FB in the association between GPR and AKI in TBI patients (Figure S2 in the **Online Supplementary Document**). Results showed that FB partially mediated this association, with a mediation effect of 4.49 × 10^−4^ (95% CI = 2.04 × 10^−4^–1.18 × 10^−3^, *P* < 0.001).

## DISCUSSION

In this work, we evaluated the association between GPR and AKI in patients with TBI. We found that elevated GPR was positively linked with the risk of occurrence in such patients. RCS regression analysis showed a potential linear correlation between the two. An exploratory subgroup analysis revealed a more pronounced positive correlation among patients with abnormal blood glucose levels. Mediation analysis indicated that FB partially mediated the association between the two variables. Sensitivity analyses confirmed that the direction and significance of the association between GPR and AKI risk remained largely consistent across varying AKI definitions and baseline creatinine criteria, alternative GPR exposure windows, adjustments for glucose/electrolyte fluctuations and insulin use, and the exclusion of patients on RRT.

Existing research indicates a significant association between GPR and the risk of death in patients with brain injury. Multiple clinical studies have confirmed that elevated GPR serves as an independent predictor of poor prognosis in individuals with TBI and cerebral haemorrhage [17,22]. Abnormally elevated GPR often reflects more severe stress-induced hyperglycaemia, and persistent hyperglycaemia can exacerbate secondary brain injury [23]. In addition, hypokalaemia, as an important component of GPR, may induce fatal arrhythmias and increase the risk of sudden death [24]. GPR abnormalities are often accompanied by more severe systemic inflammatory responses and metabolic disorders, which collectively contribute to a poor prognosis.

We herein found that GPR was strongly associated with the risk of AKI in TBI patients, which may be due to the complex interplay of multiple factors. First, the stress response after TBI leads to metabolic disturbances manifested by elevated blood glucose and disturbed potassium metabolism [25,26]. Elevated blood glucose is usually caused by increased sympathetic nerve activity and increased secretion of stress hormones. Hyperglycaemia directly damages renal tubules and renal micro vessels through oxidative stress and inflammation [23,27]. At the same time, the abnormality of potassium will also increase the burden on the kidney, especially when the renal tubular function is damaged, the excretion of potassium will further worsen the renal function [28]. Future studies should further elucidate the mechanisms linking the two and clarify the exact role of GPRs in AKI development and progression, thereby contributing to our understanding and identification of novel therapeutic targets for AKI.

The influence of GPR on AKI risk may be partially related to FB levels. First, a significantly increased GPR usually indicates a severe hyperglycaemic state. Hyperglycaemia is often accompanied by osmotic diuresis, polyuria, and fluid loss in clinical practice, which may cause negative FB in patients [29,30]. Second, hypokalaemia is closely associated with tubular dysfunction, which may impair the kidney’s concentrating ability, further exacerbating fluid imbalance [31,32]. This unstable internal environment, combined with inadequate renal blood perfusion and the risk of renal ischemia, may further increase the likelihood of developing AKI [33]. Previous studies have confirmed that FB disorders are significantly associated with increased AKI risk in critically ill patients [34,35].

While GPR had a statistically significant positive association with AKI risk in TBI patients, the absolute values of most ORs were close to 1.0, suggesting a relatively limited increase in the AKI risk by per unit rise in GPR. Nevertheless, its statistical significance reflects a stable dose-response relationship between GPR and AKI. In critically ill populations, where baseline risk was high and patients were exposed to multiple strong interventions, the effect size of any single variable in multivariate models is often conservative. However, even a small relative risk increase may translate into a considerable rise in absolute event numbers in a high-risk group, underscoring its potential clinical and public health relevance [36]. Additionally, as a ratio derived from routine, low-cost, and dynamic measurements of blood glucose and potassium [9], GPR represents an accessible indicator that can be readily integrated into existing workflows. Rather than being a strong standalone determinant, it may serve as a practical risk signal for identifying patients at higher AKI risk given its consistent association with AKI.

Notably, subgroup analyses indicated that the GPR-AKI association was more pronounced in males, patients with severe TBI (GCS = 3–8), and those with diabetes, highlighting these subgroups as particularly susceptible. Clinically, this means that persistently high GPR in these patients, even with modest effect sizes, should prompt closer monitoring of renal function, more cautious fluid management, and more prudent, earlier review of potential nephrotoxic exposures, supporting a proactive, stratified approach to care. Thus, our study does not aim to propose a single high-impact predictor, but rather to highlight a routine marker with potential clinical relevance that could be integrated into multimodal assessments to support decision-making on FO risk in patients with TBI.

There are some limitations in this study. First, the data in the MIMIC-IV database is retrospective, which may be subject to selection bias and incomplete recording, potentially impacting the accuracy and generalizability of the research findings. Second, the lack of monitoring of dynamic glucose and potassium levels in the database may not fully capture the blood glucose and potassium changes experienced by patients during their hospital stay, which could hinder a deeper investigation of the relationship between GFR and AKI. Third, TBI was defined using ICD diagnosis codes and admission GCS, without incorporating key clinical details (*e.g.* injury mechanism, neurosurgical interventions, or intracranial lesion severity), which may introduce residual confounding or measurement error in our estimates. Additionally, although models suggested that fluid balance may lie on the pathway between GPR and AKI, the observational nature of the study limits our ability to distinguish true mediation from unmeasured confounding. Thus, the FB findings are hypothesis-generating and require confirmation in more rigorous prospective studies. Finally, hyperglycaemia in GPR may reflect both injury severity and stress response. Despite adjusting for known severity indices, unmeasured confounding related to trauma severity may persist. Future studies should incorporate more granular injury biomarkers to better delineate the independent effect of GPR.

## CONCLUSIONS

We found that elevated GPR may be associated with an increased risk of AKI, with FB playing a mediating role. This discovery provides a new indicator for assessing AKI risk in patients with TBI in clinical practice. Future studies should further validate this conclusion and provide a more comprehensive understanding of GPR’s role in AKI in TBI patients, thereby offering more effective guidance for clinical treatment.

## Additional material


Online Supplementary Document
