# Supplementary material for: Association between serum glucose/potassium ratio and acute kidney injury in patients with traumatic brain injury: based on MIMIC-IV database
Source: J Glob Health. 2026 Mar 27;16:04108. doi: 10.7189/jogh.16.04108 (PMC13023681; doi:10.7189/jogh.16.04108)
Supplement: Online Supplementary Document [file jogh-16-04108-s001.pdf]

Supplement to: Chen Y, Li S, Zhang C, Zeng X. Association between serum glucose/potassium ratio and acute kidney injury in patients with traumatic brain injury: based on MIMIC-IV database. J Glob Health. 2026;16:04108.

Table S1: Results of multicollinearity test.

Variable	VIF
GPR	1.073
Sex	1.107
Race	1.029
Age	1.290
MBP	1.051
Temperature	1.079
GCS	1.027
Platelets	1.039
Sodium	1.021
RDW	1.080
Creatinine	1.454
INR	3.061
PT	3.079
Myocardial infarct	1.042
Cerebrovascular disease	1.019
Chronic kidney disease	1.200
Diabetes	1.076
Vasopression	1.068
Sepsis	1.047
Baseline eGFR	1.561
Hyperosmolar therapy	1.027
Anticoagulant	1.031
Diuretics	1.022
Glucocorticoids	1.023
Vancomycin	1.056
Fluid resuscitation	1.025

Table S2: Potential impact of baseline creatinine estimation bias on AKI classification.

Model description	N	OR (95% CI)	*p*-value
Full cohort (all patients)	1536	1.013 (1.003-1.024)	0.015
Patients with pre-ICU creatinine record	1411	1.013 (1.002-1.024)	0.020

The model was adjusted for age, race, sex, mean blood pressure, body temperature, platelet count, sodium ion concentration, mean corpuscular width, creatinine level, prothrombin time, international standardization ratio, baseline eGFR, Glasgow Coma Scale score, vasopressin, hyperosmolar therapy, anticoagulant therapy, sepsis, chronic kidney disease, myocardial infarction, cerebrovascular disease, diabetes, fluid resuscitation, diuretics, glucocorticoids, and vancomycin..

Table S3: Association of GPR with AKI stage and AKI time.

Characteristics	N (%)	OR (95% CI), *p*<br>Model 2
AKI		
AKI stage		
0	374	Ref.

**1**	**272**	**1.008 (0.998-1.023), 0.211**
**2**	**606**	**1.015 (1.004-1.028), 0.012**
**3**	**284**	**1.019 (1.005-1.034), 0.008**
**AKI time**		
**Non-AKI**	**374**	**Ref.**
**Early**	**1014**	**1.013 (1.003-1.024), 0.022**
**Late**	**148**	**1.021 (1.006-1.041), 0.023**

Table S4: Association between time-windowed mean GPR (0-72 h) and AKI in TBI patients.

**Characteristics**	**OR (95% CI),** ***p*** **Model 2**
**GPR**	
**0-24 h**	**1.019 (1.006-1.034), 0.006**
**24-48 h**	**1.015 (1.001-1.030), 0.040**
**48-72 h**	**1.026 (1.011-1.041), <0.001**

Model 2 was adjusted for age, race, sex, mean blood pressure, body temperature, platelet count, sodium ion concentration, mean corpuscular hemoglobin, creatinine level, prothrombin time, international normalized ratio, baseline eGFR, Glasgow Coma Scale score, vasopressin, hyperosmolar therapy, anticoagulant therapy, sepsis, chronic kidney disease, myocardial infarction, cerebrovascular disease, diabetes, fluid resuscitation, diuretics, glucocorticoids, and vancomycin.

Table S5: Competing risk analysis for the association between GPR and time to AKI onset.

**Characteristics**	**HR (95% CI),** ***p*** **Model 2**
**GPR**	**1.002 (1.000-1.004), 0.020**

Table S6: Comparison of AKI prediction performance between baseline GPR and 72-hour average GPR.

**Model description**	**OR (95% CI)**	***p*-value**	**AIC**	**AUC**
**GPR (preliminary model)**	**1.013 (1.003-1.024)**	**0.015**	**1682.3**	**0.645**
**Average GPR in the first 72 hours**	**1.026 (1.010-1.044)**	**0.002**	**1680.0**	**0.650**

Table S7: Association between GPR and AKI: primary model versus the model adjusted for glycemic and potassium variability.

**Model description**	**GPR OR (95% CI)**
**Preliminary model**	**1.013 (1.003-1.024)**
**Sensitivity model**	**1.012 (1.001-1.023)**

The original model was adjusted for age, race, sex, mean blood pressure, body temperature, platelet count, sodium ion concentration, mean red blood cell width, creatinine level, prothrombin time, international normalized ratio (INR), baseline estimated glomerular filtration rate (eGFR), Glasgow Coma Scale (GCS), vasopressin, hyperosmolar therapy, anticoagulant therapy, sepsis, renal disease, myocardial infarction (MI), cerebrovascular disease, diabetes mellitus, fluid resuscitation, diuretics, glucocorticoids, and vancomycin. The sensitivity model further adjusted for the coefficient of variation of blood glucose and serum potassium.

Table S8: Association between GPR and AKI with adjustment for insulin use.

Characteristics	OR (95% CI), *p*
	Model 3
GPR	1.012 (1.002-1.024), 0.039

Model 3 was adjusted for age, race, sex, mean blood pressure, body temperature, platelet count, sodium ion concentration, mean corpuscular hemoglobin, creatinine level, prothrombin time, international normalized ratio, baseline eGFR, Glasgow Coma Scale score, vasopressin, hyperosmolar therapy, anticoagulant therapy, sepsis, renal disease, myocardial infarction, cerebrovascular disease, diabetes, fluid resuscitation, diuretics, glucocorticoids, vancomycin, and insulin.

Table S9: Association of GPR with fluid overload and the AKI risk in TBI patients.

Characteristics	OR (95% CI), *p*
	Model 2
GPR	
FO	1.007 (1.001-1.014), 0.039
AKI	1.013 (1.003-1.024), 0.016

Table S10: Comparative analysis of the associations between AKI and its individual components (glucose, potassium) versus the composite ratio.

Model	Key variables OR (95% CI)	*p*-value	AIC	AUC
Only blood glucose	1.006 (1.003-1.009)	<0.001	1687.2	-
Only blood potassium	1.297 (1.085-1.564)	0.005	1700.8	-
Only GPR	1.016 (1.007-1.026)	0.001	1696.0	-
Blood glucose+Blood potassium	glucose: 1.006 (1.003-1.008)<br>potassium: 1.279 (1.066-1.549)	glucose: <0.001<br>potassium: 0.010	1682.1	0.591
Blood glucose+Blood potassium+GPR	GPR: 1.065 (1.001-1.133)<br>glucose: 0.990 (0.974-1.005)<br>potassium: 2.066 (1.267-3.549)	GPR: 0.045<br>glucose: 0.197<br>potassium: 0.006	1679.6	0.594

Figure S1.

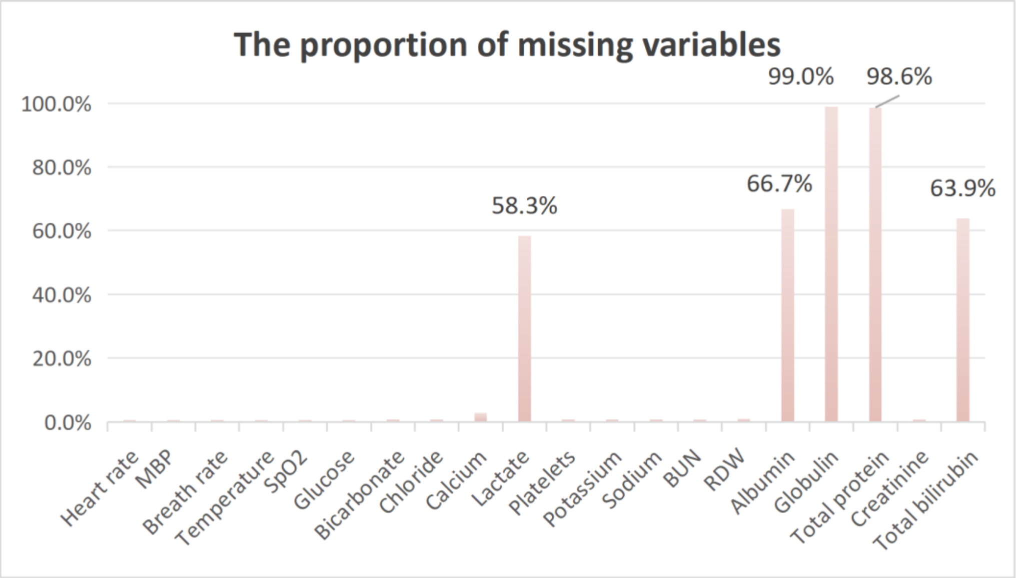

Figure S2.

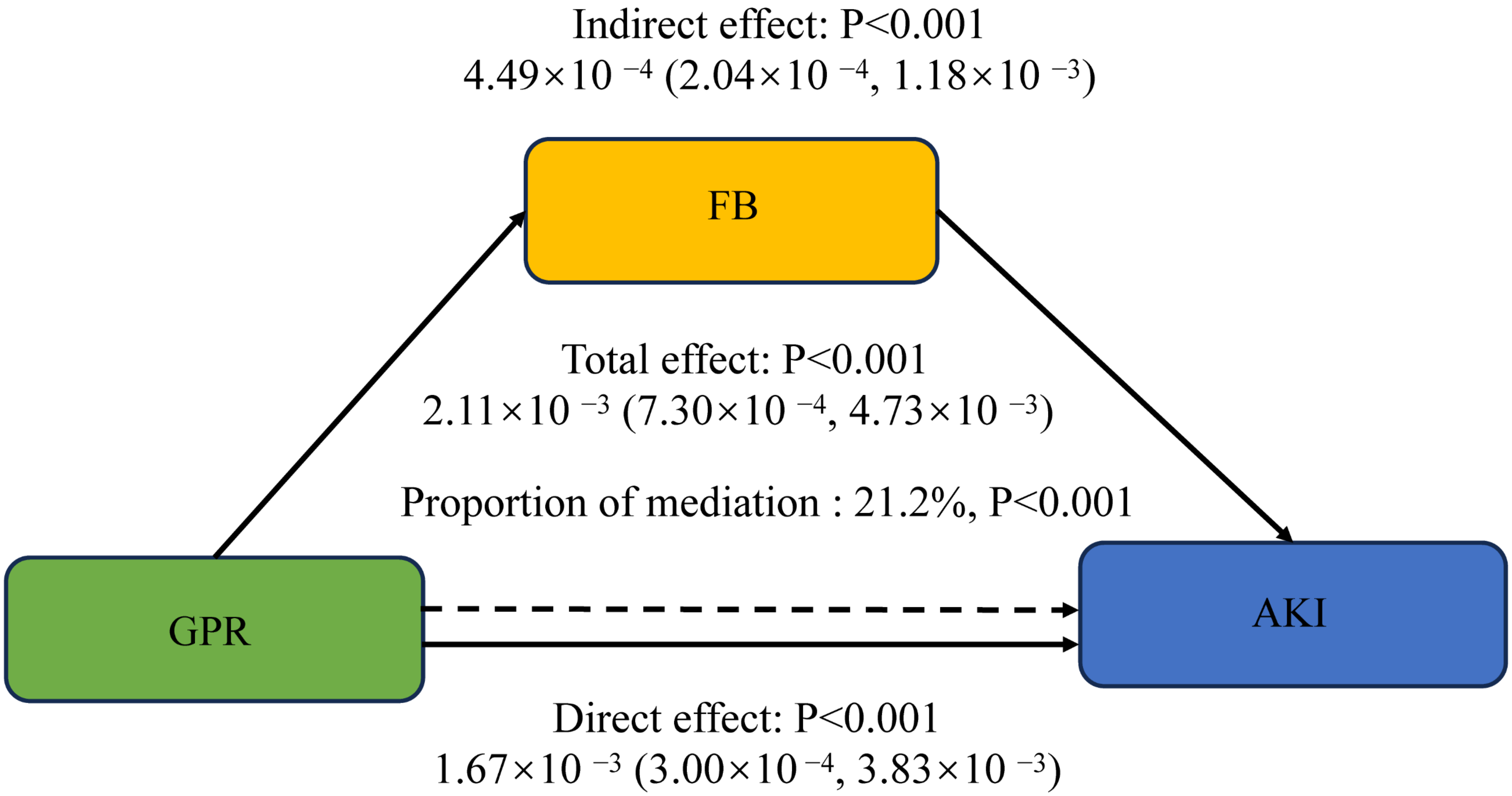
Indirect effect: P<0.001
4.49×10 −4 (2.04×10 −4, 1.18×10 −3)
FB
Total effect: P<0.001
2.11×10 −3 (7.30×10 −4, 4.73×10 −3)
Proportion of mediation : 21.2%, P<0.001
GPR
AKI
Direct effect: P<0.001
1.67×10 −3 (3.00×10 −4, 3.83×10 −3)
